# Zebularine upregulates expression of CYP genes through inhibition of DNMT1 and PKR in HepG2 cells

**DOI:** 10.1038/srep41093

**Published:** 2017-01-23

**Authors:** Kazuaki Nakamura, Kazuko Aizawa, Kyaw Htet Aung, Junji Yamauchi, Akito Tanoue

**Affiliations:** 1 Department of Pharmacology, National Research Institute for Child Health and Development, 2-10-1 Okura, Setagaya, Tokyo, 157-8535, Japan

## Abstract

Drug-induced hepatotoxicity is one of the major reasons cited for drug withdrawal. Therefore, it is of extreme importance to detect human hepatotoxic candidates as early as possible during the drug development process. In this study, we aimed to enhance hepatocyte functions such as CYP gene expression in HepG2 cells, one of the most extensively used cell lines in evaluating hepatotoxicity of chemicals and drugs. We found that zebularine, a potent inhibitor of DNA methylation, remarkably upregulates the expression of CYP genes in HepG2 cells. In addition, we revealed that the upregulation of CYP gene expression by zebularine was mediated through the inhibition of both DNA methyltransferase 1 (DNMT1) and double-stranded RNA-dependent protein kinase (PKR). Furthermore, HepG2 cells treated with zebularine were more sensitive than control cells to drug toxicity. Taken together, our results show that zebularine may make HepG2 cells high-functioning and thus could be useful for evaluating the hepatotoxicity of chemicals and drugs speedily and accurately in *in-vitro* systems. The finding that zebularine upregulates CYP gene expression through DNMT1 and PKR modulation sheds light on the mechanisms controlling hepatocyte function and thus may aid in the development of new *in-vitro* systems using high-functioning hepatocytes.

The liver is essential for maintaining normal physiology and homeostasis of the body. Among its multiple critical roles, the metabolism of chemicals, toxins and drugs by hepatocytes is especially important. Abnormality in homeostasis is often associated with hepatotoxicity^1^. Therefore, a simple and rapid method of assaying a substance’s potential for liver damage is needed to predict the toxicity and adverse effects of chemicals for pathophysiological and toxicological purposes^2^. Many preliminary hepatotoxicity studies have been carried out using *in-vivo* and/or *in-vitro* animal models, but we should not rely too heavily on such models because of species differences, animal protection concerns and model accuracy issues. Thus more reliable and more practical human *in-vitro* cell culture models are needed to improve the effectiveness with which we can evaluate the human hepatotoxicity of substances as well as reduce the number of animals used during each drug’s development process. *In-vitro* models using human cells derived from the liver, such as primary hepatocytes and hepatoma cell lines, are preferred^3^. Various sources of hepatic tissue, including whole or partial livers from organ donors or cadavers and resected liver tissue from therapeutic hepatectomies or small surgical biopsies, have been used to generate human hepatocyte cultures^4^. Yet hepatocytes originating from these sources sometimes exhibit lower cell viability in culture because they come from diseased livers (e.g., cirrhotic livers) or are damaged during the handling process. Furthermore, it is difficult to guarantee a ready supply of freshly isolated human hepatocytes, and primary human hepatocytes are known to rapidly lose cellular functions such as albumin production and their cytochrome P450 (CYP) enzymes during *in-vitro* culture.

Immortalized hepatic cell lines offer several advantages over primary hepatocytes in terms of handling and use, including easier maintenance, cryopreservation and revival; lower cost and less time required for handling; less need for specialized techniques; and genetic uniformity of the resulting sample cultures. The immortalized hepatic cell line known as HepG2, derived from human hepatocellular carcinomas and retaining many metabolic functions of the human liver^5^,^6^, is one of the most extensively used cell lines in the evaluation of the toxicity of chemicals and drugs^7^. Compared to primary human hepatocytes, however, HepG2 cells express lower levels of cytochrome P450 (CYP) enzymes, which metabolize drugs in hepatocytes^3^.

Gene expression in eukaryotic cells is controlled by several factors including epigenetic mechanisms. At least three systems are involved in initiating and sustaining epigenetic change, including DNA methylation, histone modification and non-coding RNA (ncRNA)-associated gene silencing^8^. In HepG2 cells, epigenetic factors are known to play a role in regulating the expression of several genes involved in essential liver processes such as xenobiotic metabolism and steroid biosynthesis. It is known that DNA methylation plays a role much larger than that of histone deacetylation in regulating gene expression in HepG2 cells^9^. Although the hypermethylation of promoter CpG islands can result in gene silencing, which consequently leads to suppressed cellular functions, epigenetic changes such as DNA methylation are pharmacologically reversible. DNA methylation is inhibited by demethylating agents such as 5-aza-2′-deoxycytidine (5-aza-dC). 5-aza-dC exerts its demethylating function by sequestering DNA methyltransferase 1 (DNMT1) to 5-aza-dC-substituted DNA via the irreversible binding of cysteine in the catalytic domain of DNMT1 to the 6 position of the cytidine ring^10^,^11^. This decreases the cellular concentration of DNMT1 in the course of successive cell divisions^12^,^13^,^14^. Many genes in HepG2 cells are either turned on or upregulated by 5-aza-dC^9^.

Zebularine (1-(β-d-ribofuranosyl)-1, 2-dihydropyrimidin-2-one), which is a nucleoside analog of cytidine, is a second-generation, highly stable hydrophilic inhibitor of DNA methylation^15^. It acts primarily as a trap for DNMT proteins by forming tight covalent complexes between DNMT proteins and zebularine-substitute DNA^16^. Several studies have demonstrated that the rate of methylation of DNA containing incorporated zebularine is lower than that of unmodified DNA^16^,^17^. Although inhibitors of DNA methylation rapidly reactivate the expression of genes that have undergone epigenetic silencing^8^, little is known about the impact of zebularine on gene expression in HepG2 cells or how it can be used in *in-vitro* assays.

In the present study, we investigated the effect of zebularine on CYP gene expression and sensitivity for hepatotoxicity in HepG2 cells. We found that zebularine upregulates CYP gene expression and that this effect is mediated through the inhibition of DNMT1 and double-stranded RNA-dependent protein kinase (PKR); this is a novel finding about the mechanism of CYP gene expression. In addition, we found that HepG2 cells treated with zebularine are highly susceptible to xenobiotic cytotoxicity compared with controls. These results will be useful for generating high-functioning HepG2 cells and may contribute to the development of a simple and rapid method of assaying a substance’s potential for liver damage.

## Results

### Zebularine upregulates expression of CYP genes in HepG2 cells

**Figure 1:**
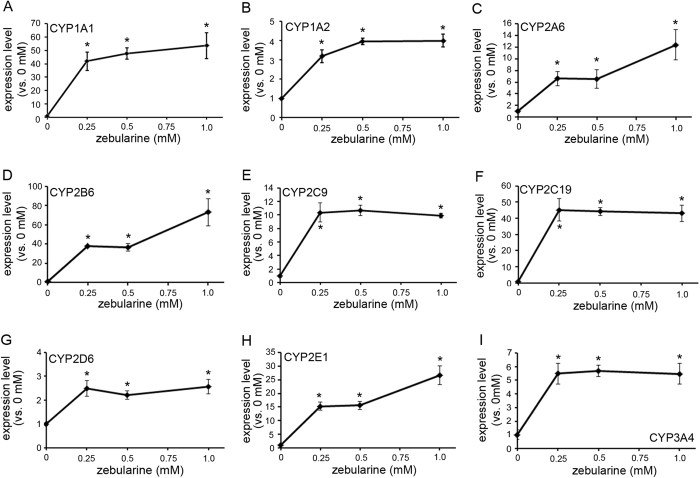
The effect of zebularine on CYP gene expression in HepG2 cells. (**A**–**I)** HepG2 cells were exposed to zebularine for 72 h. Expression levels of CYP1A1 (**A**), 1A2 (**B**), 2A6 (**C**), 2B6 (**D**), 2C9 (**E**), 2C19 (**F**), 2D6 (**G**), 2E1 (**H**) and 3A4 (**I**) were examined by qRT-PCR. The comparative threshold cycle (Ct) method was used to determine the relative ratio of expression for each gene, which was corrected against HPRT1. Each data point represents the mean ± SEM of the values obtained from three independent experiments. The expression level measured after exposure to 0 mM zebularine was considered to be 1.0 expression. *p < 0.05, compared to 0 mM.

**Figure 2:**
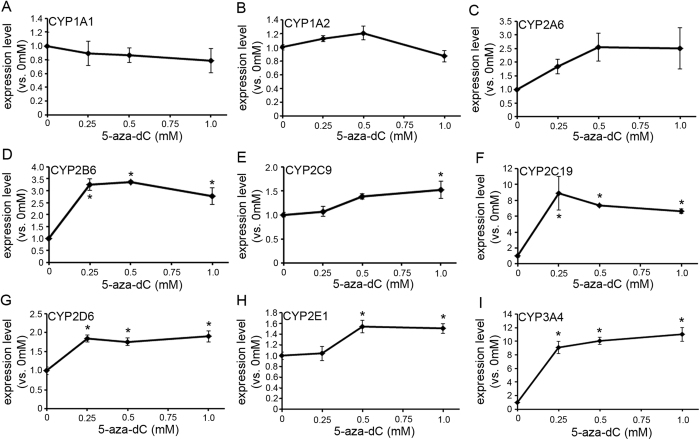
The effect of 5-aza-dC on CYP gene expression in HepG2 cells. (**A**–**I**) HepG2 cells were exposed to 5-aza-dC for 72 h. Expression levels of CYP1A1 (**A**), 1A2 (**B**), 2A6 (**C**), 2B6 (**D**), 2C9 (**E**), 2C19 (**F**), 2D6 (**G**), 2E1 (**H**) and 3A4 (**I**) were examined by qRT-PCR. The comparative threshold cycle (Ct) method was used to determine the relative ratio of expression for each gene, which was corrected against HPRT1. Each data point represents the mean ± SEM of the values obtained from three independent experiments. For each gene, the expression level detected in cells treated with 0 mM 5-aza-dC was considered to be 1.0 expression. *p < 0.05, compared to 0 mM.

**Figure 3:**
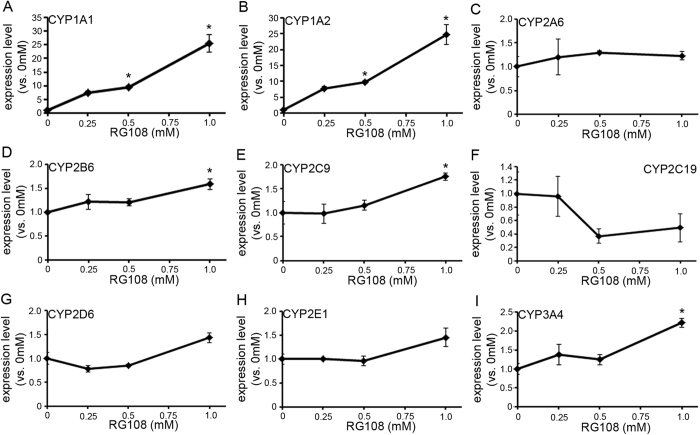
The effect of RG108 on CYP gene expression in HepG2 cells. (**A**–**I)** HepG2 cells were exposed to RG108 for 72 h. Expression levels of CYP1A1 (**A**), 1A2 (**B**), 2A6 (**C**), 2B6 (**D**), 2C9 (**E**), 2C19 (**F**), 2D6 (**G**), 2E1 (**H**) and 3A4 (**I**) were examined by qRT-PCR. The comparative threshold cycle (Ct) method was used to determine the relative ratio of expression for each gene, which was corrected against HPRT1. Each data point represents the mean ± SEM of the values obtained from three independent experiments. For each gene, the expression level detected in cells treated with 0 mM RG108 was considered to be 1.0 expression. *p < 0.05, compared to 0 mM.

Among the CYPs, the forms most highly expressed in the liver are CYPs 3A4, 2C9, 2C8, 2E1 and 1A2, while CYPs 2A6, 2D6, 2B6, 2C19 and 3A5 are less abundant and CYPs 2J2, 1A1 and 1B1 are mainly expressed extrahepatically^18^. In the present study, we examined the expression patterns of CYPs 1A1, 1A2, 2A6, 2B6, 2C9, 2C19, 2D6, 2E1 and 3A4, which are collectively responsible for the biotransformation of most foreign substrates including 99.5% of those in clinical use. We treated HepG2 cells with zebularine for 72 h, then examined CYP gene expressions. Interestingly, zebularine upregulated the expression levels of all nine of the CYP genes examined in this study (Fig. 1), though there was some variability in the strength of this effect. CYP1A1 (Fig. 1A), 2B6 (Fig. 1D), 2C19 (Fig. 1F) and 2E1 (Fig. 1H) were classified into the strongly upregulated (>20 fold) group; CYP2A6 (Fig. 1C) and 2C9 (Fig. 1E) were classified into the moderately upregulated (>10 fold) group; and CYP1A2 (Fig. 1B), 2D6 (Fig. 1D) and 3A4 (Fig. 1I) were classified into the slightly upregulated (<10 fold) group. In order to examine the effective duration of zebularine, we removed zebularine from culture medium for 72 h after a 72 h period of zebularine exposure, then examined CYP gene expressions. We found that almost all CYP genes’ expression levels were decreased after 72 h without zebularine (Supplementary Fig. S1) compared with just after zebularine treatment (Fig. 1). These results suggest that upregulation of CYP gene expression by zebularine can be reversed by removing zebularine from the culture media.

Because zebularine upregulated CYP gene expressions in HepG2 cells, we examined the effect of zebularine in human primary hepatocytes. We treated primary human hepatocytes with zebularine for 72 h, then examined CYP gene expressions. Unfortunately, we found that CYP gene expression was not affected by zebularine in human primary hepatocytes (Supplementary Fig. S2).

### Zebularine specifically upregulates CYP gene expression relative to other DNMT inhibitors

Since zebularine affected the expression of CYP genes in HepG2 cells, we studied the effects of two other DNMT inhibitors, 5-aza-dC and RG108. 5-aza-dC is reported to upregulate the expression of CYP3A genes in HepG2 cells^9^, but there is no evidence regarding whether DNMT inhibition affects any other CYP genes. RG108 is a novel non-nucleoside DNMT inhibitor that blocks the enzyme active site by directly interacting with DNMT. We treated HepG2 cells with 5-aza-dC or RG108 for 72 h and examined CYP gene expressions. The expression levels of most of the examined CYP genes were significantly upregulated by treatment with 5-aza-dC (Fig. 2) and RG108 (Fig. 3), but zebularine had a stronger upregulating effect, suggesting that zebularine is the most effective among these three DNMT inhibitors.

In addition to inhibiting DNMT, zebularine is also known to inhibit cytidine deaminase^19^. Therefore we investigated whether the inhibition of cytidine deaminase activity could be involved in the upregulation of CYP gene expression by zebularine. To test the possible involvement of cytidine deaminase in regulating CYP gene expression, we used tetrahydrouridine, a well-known and potent inhibitor of cytidine deaminase that competitively blocks the enzyme’s active site more effectively than intrinsic cytidine does^20^,^21^. We treated HepG2 cells with tetrahydrouridine for 72 h, then examined CYP gene expression levels. We found that CYP gene expression was not affected by tetrahydrouridine treatment (Supplementary Fig. S3). These results suggest that zebularine may upregulate CYP gene expression levels by inhibiting DNMT activity but not cytidine deaminase activity.

### DNMT1 and PKR are involved in upregulation of CYP genes by zebularine treatment in HepG2 cells

**Figure 4:**
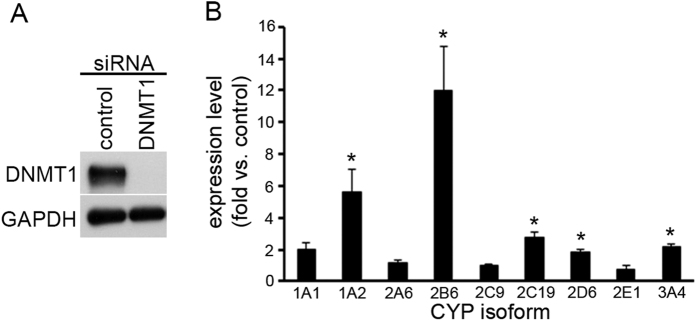
The effect of knockdown of DNMT1 on CYP gene expression in HepG2 cells. (**A)** HepG2 cells were transfected with siRNA against DNMT1 or negative control. After 72 h transfection, the cells were harvested and western blot analysis was performed to measure the DNMT1 protein level. GAPDH was used as a loading control. (**B)** Expression levels of CYP1A1, 1A2, 2A6, 2B6, 2C9, 2C19, 2D6, 2E1 and 3A4 in HepG2 cells transfected with siRNA against DNMT1 were examined by qRT-PCR. The comparative threshold cycle (Ct) method was used to determine the relative ratio of expression for each gene, which was corrected against HPRT1. Each data point represents the mean ± SEM of the values obtained from three independent experiments. The expression level of HepG2 transfected with negative control was considered to be 1.0 expression. *p < 0.05, compared to control HepG2 cells.

In our previous study^22^, we reported that zebularine treatment downregulated the expression of DNMT1 protein in HepG2 cells, implying that zebularine can upregulate the expression levels of CYP genes through suppressing the expression of DNMT1. To examine the action mechanism by which zebularine upregulates the expression levels of CYP genes, we knocked down DNMT1 gene expression in HepG2 cells and examined the expression levels of CYP genes. Transfection of siRNA against DNMT1 reduced the expression level of DNMT1 protein after 72 h transfection (Fig. 4A), but knockdown of DNMT1 did not upregulate the expressions of CYP genes (Fig. 4B) as effectively as zebularine treatment did (Fig. 1). The expression levels of several CYP genes, including CYP1A2, CYP2B6, CYP2C19, CYP2D6, and CYP3A4, were significantly upregulated, but the remaining genes were not affected by the knockdown of DNMT1. Furthermore, the upregulation of gene expression resulting from knockdown of DNMT1 was less powerful than that resulting from zebularine treatment (Fig. 1). This suggests that zebularine can partially upregulate the expression levels of CYP genes through suppressing the expression of DNMT1, but that other mechanism(s) in addition to DNMT1 inhibition could be involved in the upregulation of CYP genes induced by zebularine treatment.

**Figure 5:**
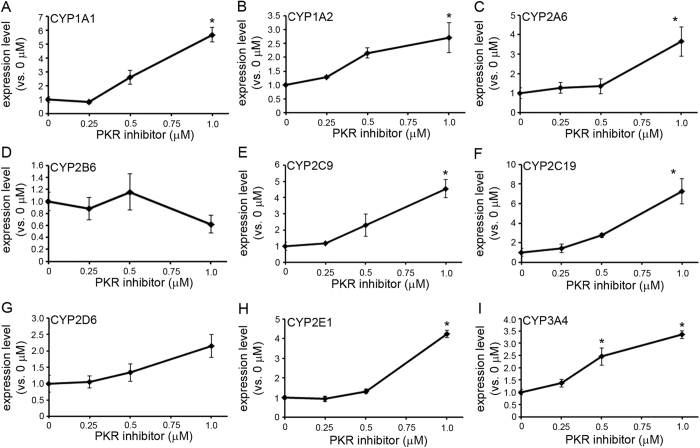
The effect of PKR inhibitor on CYP gene expression in HepG2 cells. (**A**–**I)** HepG2 cells were exposed to PKR inhibitor for 72 h. Expression levels of CYP1A1 (**A**), 1A2 (**B**), 2A6 (**C**), 2B6 (**D**), 2C9 (**E**), 2C19 (**F**), 2D6 (**G**), 2E1 (**H**) and 3A4 (**I**) were examined by qRT-PCR. The comparative threshold cycle (Ct) method was used to determine the relative ratio of expression for each gene, which was corrected with HPRT1. Each data point represents the mean ± SEM of the values obtained from three independent experiments. The expression level measured after exposure to 0 μM PKR inhibitor was considered to be 1.0 expression. *p < 0.05, compared to 0 μM.

In order to investigate these possible mechanism(s), we assessed the effect of PKR inhibition on CYP gene expression, since our previous study had revealed that zebularine inhibits not only DNMT1 but also PKR in HepG2 cells^22^. We treated HepG2 cells with a PKR inhibitor for 72 h and investigated the expression levels of CYP genes. Treatment with the PKR inhibitor upregulated the expression levels of most of the examined genes, including CYP1A1 (Fig. 5A), CYP1A2 (Fig. 5B), CYP2A6 (Fig. 5C), CYP2C9 (Fig. 5E), 2C19 (Fig. 5F), CYP2E1 (Fig. 5H) and CYP3A4 (Fig. 5I), but other genes such as CYP2B6 (Fig. 5D) and CYP2D6 (Fig. 5G) were not affected. Furthermore, upregulation of gene expression levels induced by the PKR inhibitor was less powerful than that induced by zebularine (Fig. 1), as observed in the case of DNMT1 knockdown (Fig. 4). These results suggest that the inhibition of PKR activity as well as DNMT1 can upregulate the expression of CYP genes to some degree, but not as thoroughly as zebularine treatment can.

**Figure 6:**
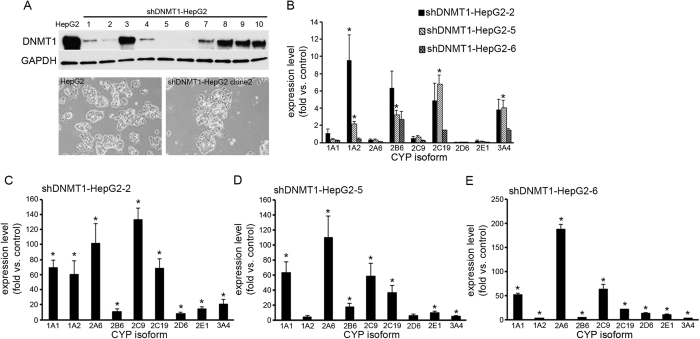
The effect of combined DNMT1 and PKR inhibition on CYP gene expression in HepG2 cells. (**A)** shDNMT1 was stably transfected into HepG2 cells and shDNMT1-HepG2-2, -5 and -6 cells showed downregulation of DNMT1 protein level. (**B)** Expression levels of CYP1A1, 1A2, 2A6, 2B6, 2C9, 2C19, 2D6, 2E1 and 3A4 were examined in shDNMT1-HepG2-2, -5 and -6 cells by qRT-PCR. The expression level observed in parent cells was considered to be 1.0 expression. (**C**–**E)** shDNMT1-HepG2-2 cells (**C**), shDNMT1-HepG2-5 cells (**D**) and shDNMT1-HepG2-6 cells (**E**) were exposed to 1000 μM PKR inhibitor for 72 h, and expression levels of CYP1A1, 1A2, 2A6, 2B6, 2C9, 2C19, 2D6, 2E1 and 3A4 were examined by qRT-PCR. The expression level of parent HepG2 cells without PKR inhibitor treatment was considered to be 1.0 expression. The comparative threshold cycle (Ct) method was used to determine the relative ratio of expression for each gene, which was corrected against HPRT1. Each data point represents the mean ± SEM of the values obtained from three independent experiments. *p < 0.05, compared to parent HepG2 cells.

Next, we examined the effect of the combined inhibition of DNMT1 and PKR on the expression of CYP genes, since the inhibition of either DNMT1 or PKR activity alone resulted in only partial upregulation. To evaluate the effect of the combined inhibition of both genes, we generated mutant HepG2 cells with downregulated DNMT1 activity through the stable expression of shRNA against DNMT1, and treated these mutant cells with the PKR inhibitor for 72 h. After transfecting shRNA against DNMT1, we established three mutant clones of shDNMT1-HepG2 cells (shDNMT1-HepG2-2, -5 and -6 clones), all with downregulated DNMT1 expression (Fig. 6A). There were no morphological differences among these shDNMT1-HepG2 cells, and all were similar to the parent HepG2 cells (Fig. 6A). We first investigated the expression levels of CYP genes in all three shDNMT1-HepG2 clones, and found that some CYP genes’ expression levels were upregulated compared to those in the parent cells (Fig. 6B), though the degree of CYP gene upregulation was not comparable to that induced by zebularine (Fig. 1), but rather similar to that seen in cells transfected with siRNA against DNMT1 (Fig. 4). We then investigated the expression levels of CYP genes in each of the three shDNMT1-HepG2 clones after treatment with the PKR inhibitor, and found that all of the examined gene expressions were remarkably increased in shDNMT1-HepG2-2 and -6 cells, and that most of them were significantly increased in shDNMT1-HepG2-5 cells (Fig. 6C–E). The upregulated gene expression levels observed in these cells were similar to those observed in cells treated with zebularine (Fig. 1). Thus, the combined inhibition of DNMT1 and PKR can induce an upregulation of CYP gene expression comparable to that induced by zebularine treatment, implying that zebularine upregulates CYP gene expression through the inhibition of both DNMT1 and PKR.

**Figure 7:**
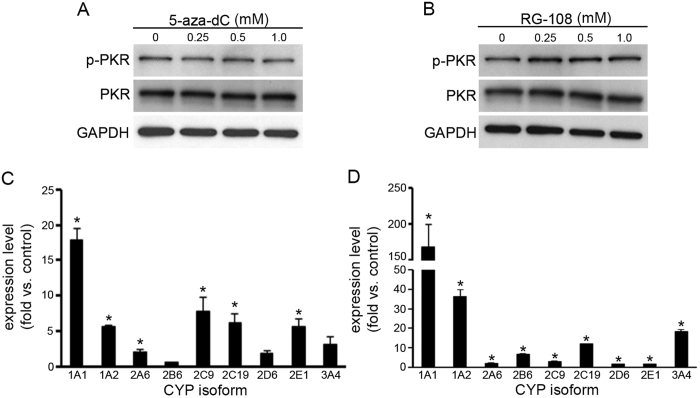
The effect of the combination of either 5-aza-dC or RG108 and PKR inhibitor on CYP gene expression in HepG2 cells. (**A,B)** The phosphorylation and expression of PKR after 5-aza-dC (**A**) or RG108 (**B**) treatment for 72 h at different concentrations. After treatment, the cells were harvested and western blot analysis was performed to detect the phosphorylated and total PKR protein levels. GAPDH was used as a loading control. A typical image is shown and the experiment was performed independently three times under each condition. (**C,D)** Expression levels of CYP1A1, 1A2, 2A6, 2B6, 2C9, 2C19, 2D6, 2E1 and 3A4 were examined in HepG2 cells exposed to a combination of PKR inhibitor and either 5-aza-dC (**C**) or RG108 (**D**). After 72 h exposure, expression levels of CYPs were examined by qRT-PCR. The comparative threshold cycle (Ct) method was used to determine the relative ratio of expression for each gene, which was corrected against HPRT1. Each data point represents the mean ± SEM of the values obtained from three independent experiments. The expression levels measured in control HepG2 cells were considered to be 1.0 expression. *p < 0.05, compared to control HepG2 cells.

**Figure 8:**
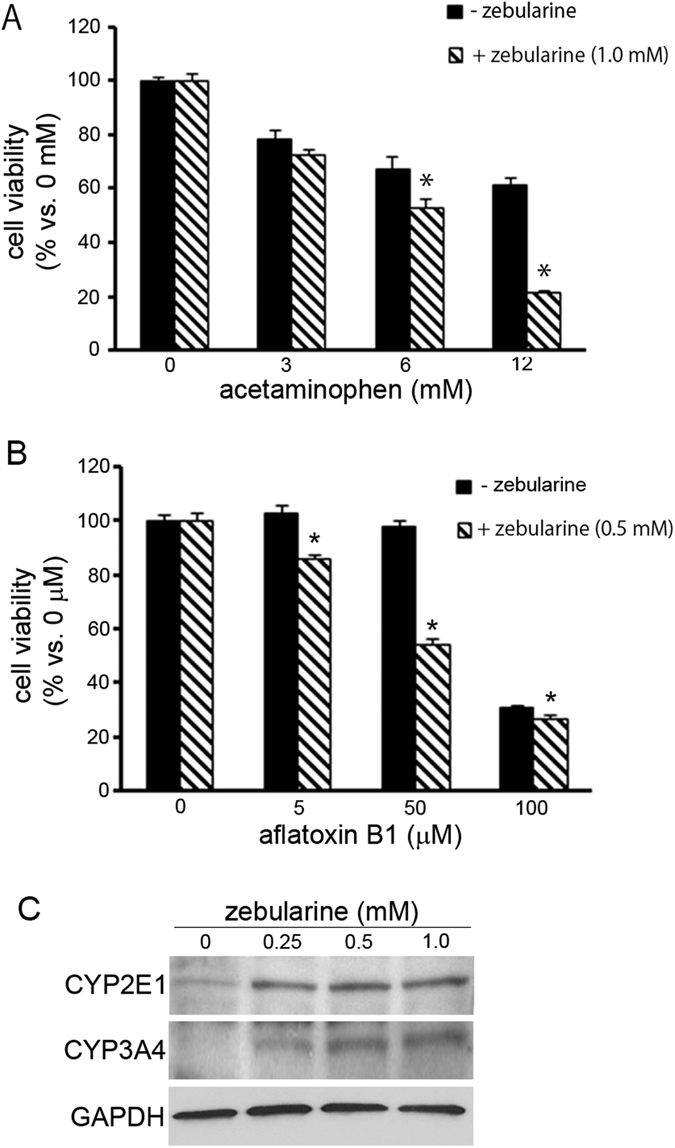
The effect of zebularine treatment on evaluation of drug toxicity. (**A,B)** Comparison of cell viability with and without zebularine. After pretreatment with zebularine for 72 h, cells were exposed to acetaminophen (**A**) or aflatoxin B1 (**B**) for the next 72 h and then examined for cell viability. Each data point represents the mean ± SEM of the values obtained from three independent experiments. Zebularine-treated and untreated cell cultures exposed to 0 mM acetaminophen or 0 μM aflatoxin B1 were considered to be 100% viable. *p < 0.05, compared to the untreated (-zebularine) group. (**C**) Analysis of CYP2E1 and CYP3A4 protein levels after exposure to zebularine. After 72 h zebularine exposure, the cells were harvested and western blot analysis was performed to measure the CYP2E1 or 3A4 protein level. GAPDH was used as a loading control.

One significant difference among the three DNMT1 inhibitors was that 5-aza-dC and RG108 could not upregulate the expression of CYP genes in HepG2 cells as effectively as zebularine could (Figs 2 and 3). To analyze the mechanism(s) underlying this difference in the drugs’ effects on HepG2 cells, we examined whether 5-aza-dC and RG108 could inhibit PKR to the same degree that zebularine can. We treated HepG2 cells with 5-aza-dC or RG108 and analyzed the phosphorylation and expression of PKR. In this study, we found that neither 5-aza-dC nor RG108 treatment could affect PKR phosphorylation or PKR protein level (Fig. 7A,B), while it is known that zebularine treatment downregulates PKR phosphorylation and PKR protein level in HepG2 cells^22^. These findings indicate that 5-aza-dC and RG108 affect DNMT1 activity but not PKR. Therefore, we next examined whether treatment with a PKR inhibitor combined with 5-aza-dC or RG108 could upregulate CYP gene expression in HepG2 cells more effectively than treatment with 5-aza-dC or RG108 alone could. Although the expression levels of CYP2B6 and 3A4 after treatment with a combination of 5-aza-dC and PKR inhibitor (Fig. 8C) are no more than half of those after treatment with 5-aza-dC alone (Fig. 2), almost all of the tested CYP genes were upregulated more effectively by 5-aza-dC (Fig. 7C) or RG108 (Fig. 7D) combined with PKR inhibitor than by 5-aza-dC (Fig. 2) or RG108 alone (Fig. 3). These results suggest that inhibition of PKR in addition to DNMT1 is critical for effective upregulation of CYP genes in HepG2 cells.

### HepG2 cells treated with zebularine are useful for evaluating xenobiotic cytotoxicity

HepG2 cells with upregulated CYP gene expression could potentially become a valuable tool for evaluating xenobiotic cytotoxicity *in vitro*. To test HepG2 cells treated with zebularine as a tool for this purpose, the toxicity of acetaminophen (APAP) and aflatoxin B1, both of which are mediated by the formation of toxic metabolites by CYPs, was evaluated in cells treated with zebularine. The toxicity of APAP was much greater in cells treated with 1.0 mM zebularine than in control (untreated) cells after 72 h (Fig. 8A). Viability rates after 6 mM and 12 mM APAP exposure were 67.0 ± 2.7% and 61.0 ± 2.7%, respectively, in control cells, but only 52.8 ± 3.3% and 21.2 ± 0.6%, respectively, in cells treated with zebularine. The toxicity of aflatoxin B1 was also much greater in cells treated with 0.5 mM zebularine than in control cells after 72 h treatment (Fig. 8B). The zebularine-treated group had a higher sensitivity for detecting the toxicity of aflatoxin B1 than the control group had. These results indicate that HepG2 cells treated with zebularine have a higher sensitivity for evaluating xenobiotic cytotoxicity compared with untreated cells.

It is known that APAP and aflatoxin B1 are metabolized by CYP2E1 and CYP3A4, respectively. We examined the levels of these proteins after zebularine exposure and found that zebularine enhanced the levels of both (Fig. 8C). These results suggest that zebularine upregulates not only CYP gene expression but also protein levels, and also enhances CYP activities, giving HepG2 cells higher sensitivity for evaluating xenobiotic cytotoxicity.

## Discussion

In this study, we found that zebularine can upregulate CYP genes and induce higher sensitivity for evaluating xenobiotic cytotoxicity in HepG2 cells. These effects of zebularine are mediated through inhibition of DNMT1 activity, but not through inhibition of cytidine deaminase activity. Furthermore, zebularine simultaneously inhibited PKR activity, which is critical to the upregulation of CYP gene expression. We also found that zebularine upregulated the expressions of CYP genes more potently than 5-aza-dC or RG108 did, due to the differences between the actions of zebularine and those of 5-aza-dC and RG108; zebularine inhibits both DNMT1 and PKR, whereas 5-aza-dC and RG108 inhibit DNMT1 but not PKR. This shows that it is important to suppress both DNMT1 and PKR activities in order to effectively upregulate the expression of CYP genes in HepG2 cells.

By analyzing the mechanism by which zebularine upregulates CYP gene expression in HepG2 cells, we found that expression levels of CYP genes were increased not only by the inhibition of DNMT1 activity but also by the inhibition of PKR activity. This is a novel function of PKR indicating that PKR as well as DNMT1 plays a critical role in regulating CYP gene expression, although the mechanism(s) underlying the regulation of CYP genes by PKR remain to be clarified. PKR has been shown to play a variety of important roles in the regulation of translation, transcription, and signal transduction pathways. PKR is activated by autophosphorylation in response to specific stresses, and leads to the phosphorylation of eukaryotic initiation factor 2 (eIF2) α, impairing its activity, which results in the inhibition of protein synthesis and apoptosis induction^23^. In addition to its translational regulatory function, PKR has a role in signal transduction and transcriptional control through the inhibition of κB (IκB)/nuclear factor κB (NF-κB) pathways^24^,^25^. Furthermore, PKR is involved in various other pathways that activate and engage a number of transcription factors controlling the expression of multiple genes, including interferon regulatory factor 1 (IRF-1), signal transducers and activators of transcription factors (STATs), mitogen-activated protein kinases (MAPKs), and p53^23^,^26^,^27^,^28^,^29^. PKR can directly stimulate cell growth through NF-κB pathways and the p38 MAPK^30^,^31^. On the other hand, it is known that cell functions in hepatocytes are inhibited when cell growth is promoted. Henkens *et al*. reported that cell functions including albumin secretion, urea production and the expression of CYP1A1 and CYP2B1 were suppressed in cultured primary hepatocytes while the cell cycle was progressing, suggesting that cell cycle inhibition may be crucial to the maintenance of primary hepatocytes’ functions during culture^32^. In our previous report, we found that zebularine inhibited cell proliferation at least in part through the suppression of PKR in HepG2 cells^22^. Taken together, the available evidence suggests that PKR might trigger the promotion of cell proliferation through the NF-κB or p38 MAPK pathways, resulting in inhibition of CYP gene expression in HepG2 cells. On the other hand, the expression levels of CYP2B6 and 3A4 after treatment with a combination of 5-aza-dC and PKR inhibitor are no more than half those after treatment with 5-aza-dC alone. These results suggest that the CYP gene expression mechanisms differ among CYP genes and that the combination of 5-aza-dC and PKR inhibitor is not always effective on CYP2B6 and 3A4 expression. Thus, the mechanism of CYP gene expression remains a matter for further research in HepG2 cells. To clarify the mechanism(s) by which zebularine upregulates CYP gene expression in HepG2 cells, further studies, including some on the relevant functions of PKR, will be needed.

In contrast to HepG2 cells, zebularine did not affect CYP gene expression levels in human primary hepatocytes. Although the precise reason why zebularine has no effect on CYP gene expression levels in primary hepatocytes remains unknown, it is possible that zebularine is metabolized by primary hepatocytes, which have relatively high drug metabolic activities, so that it cannot exert its effect. In this study, we did not examine whether the findings related to the regulation of CYP gene expressions by DNMT1 and PKR could also be applied to human hepatocytes. Future studies using knockdown of both DNMT1 and PKR in primary hepatocytes will reveal more detailed knowledge of the regulation of CYP gene expression by DNMT1 and PKR.

Because HepG2 cells express much lower levels of CYP genes than do human hepatocytes *in vivo*, cytotoxicity assays using HepG2 cells are considered to be less sensitive than those using human hepatocytes. To test whether zebularine treatment may enable us to overcome this limitation of HepG2 cells, we tested the cytotoxicity of APAP and aflatoxin B1, both of which are known to be hepatotoxic *in vivo*, in zebularine-treated cells. Cell viabilities after exposure to APAP or aflatoxin B1 were significantly lower in HepG2 cells treated with zebularine than in untreated cells, indicating that treated cells are more sensitive to the cytotoxicity of APAP or aflatoxin B1 than untreated cells. In the case of APAP, the hepatotoxicity of the drug is known to be associated with *N*-acetyl-*p*-benzoquinoneimine (NAPQI), a reactive metabolite of CYP2E1^33^, and unrepaired damage to DNA^34^,^35^. APAP is metabolized into NAPQI by CYP2E1, and the metabolized NAPQI exerts the cytotoxic effect. The expression level of CYP2E1 is 10- to 20-fold higher in cells treated with zebularine than in untreated cells, and the CYP2E1 protein level is also upregulated by zebularine, allowing the metabolism of a greater quantity of APAP to NAPQI, and consequently more noticeable cytotoxic effects. Aflatoxin B1 is also known to be hepatotoxic *in vivo*. The drug is metabolized into aflatoxin B1-8, 9-epoxide by CYP3A4, and aflatoxin B1–8, 9-epoxide exerts the toxic effect^36^. As in the case of APAP, the increases in expression and protein levels of CYP3A4 that result from zebularine treatment make the HepG2 cell assay more sensitive to the cytotoxicity of aflatoxin B1. Thus zebularine-induced upregulation of CYP genes in HepG2 cells is expected to enable the development of more sensitive *in-vitro* assays.

High-functioning hepatocytes have been proposed for various applications including bioartificial livers and liver tissue engineering as well as an *in-vitro* model for studies of drug metabolism and hepatotoxicity. Accordingly, there have been several reports about the development of *in-vitro* culture systems for high-functioning hepatocytes^37^. Spheroid culture of hepatocytes, formed through the rearrangement and compaction of cell aggregates of liver-derived cells including primary hepatocytes or cell lines, yields much higher levels of liver-specific functions than those seen in monolayer culture due to the multiple strong similarities between conditions in spheroid cultures and real tissues, although limitations with respect to the diffusion of oxygen and nutrients into the centers of large spheroids of hepatocytes should be considered^38^. We also recently reported that primary human hepatocytes as well as HepG2 cells can express higher cell functions when grown in hepatocyte spheroid culture^39^,^40^. In the present study, however, we developed another high-functioning hepatocyte culture system that uses drug treatment or gene knockdown. In this system, the effects of zebularine on CYP gene expression can be reversed by removing zebularine from the culture media. This means that HepG2 potential can be arbitrarily controlled with zebularine treatment. On the other hand, stable gene knockdown of DNMT1 and PKR may enable the continual upregulation of CYP gene expression in HepG2 cells. Considered together, these studies hint that chemical induction through drug treatment, gene knockdown of relevant factors, and spheroid culture, either alone or in combination, could result in more highly functional hepatocytes, and that the identification of an ideal combination of conditions could contribute to the development of improved *in-vitro* cytotoxicity assays and bioartificial livers. Thus our present study has shed new light on the potential of zebularine, along with spheroid culture, as a tool for the enhancement of hepatocyte function.

In summary, we found that zebularine upregulates CYP gene expression through the inhibition of both DNMT1 and PKR in HepG2 cells. Our results suggest a novel regulatory mechanism of CYP gene expression. As HepG2 cells are one of the most extensively used cell lines in evaluating the toxicity of chemicals and drugs, high-functioning HepG2 cells are very valuable for *in-vitro* assays, and zebularine treatment or specific inhibition of DNMT1 and PKR in HepG2 cells can become a useful means of generating these cells.

## Materials and Methods

### Cell culture

HepG2 cells (JCRB1054) were purchased from the Health Science Research Resources Bank (Japan Health Sciences Foundation, Osaka, Japan) and maintained at 37 °C under an atmosphere of 95% air and 5% CO_2_ in Dulbecco’s modified Eagle’s medium (DMEM) containing 10% fetal bovine serum (FBS), 100 U/ml penicillin, and 100 μg/ml streptomycin. Cells were immersed in a culture medium containing the indicated concentrations of zebularine, 5-Aza-2′-deoxycytidine (5-aza-dC), 2-1,3-dioxo-1,3-dihydro-2H-isoindol-2-yl)-3-(1H-indol-3-yl) propionic acid (RG108) or PKR inhibitor. Zebularine (Wako Pure Chemical Industries, Osaka, Japan) was dissolved in distilled water as a stock solution. 5-Aza-2′-deoxycytidine, RG108 (Wako Pure Chemical Industries), PKR inhibitor (Merck Millipore, Tokyo, Japan), acetaminophen (Sigma-Aldrich Japan, Tokyo, Japan) and aflatoxin B1 (Wako Pure Chemical Industries) were each dissolved in DMSO as a stock solution.

### Quantitative real-time PCR analysis

Quantitative reverse transcription-polymerase chain reaction assay (qRT-PCR) was performed to measure the mRNA expression levels of CYPs. Briefly, total RNA was extracted using RNeasy (QIAGEN, Tokyo, Japan) according to the manufacturer’s instructions. Total RNA was reverse-transcribed with a first-strand cDNA synthesis kit (TAKARA, Shiga, Japan) using oligo-dT primers. Premix Ex Taq (TAKARA) and TaqMan gene expression assays (Applied Biosystems, Life Technologies Japan, Tokyo, Japan) were used for the real-time quantitative polymerase chain reactions; amplifications were performed with a Thermal Cycler Dice Real Time System (TAKARA). The comparative threshold cycle (Ct) method was used to determine the relative ratio of expression for each gene, which was corrected with human hypoxanthine phosphoribosyltransferase 1 (HPRT1). The TaqMan assay numbers are listed in Supplementary Table S1.

### Immunoblot

Cells were lysed in lysis buffer and stored at −80 °C until use. After centrifugation, aliquots of the supernatants were subjected to SDS-PAGE. The electrophoretically separated proteins were transferred to PVDF membranes, blocked, and immunoblotted with anti-CYP2E1 (CR3271), CYP3A4 (CR3340) (Enzo Life Sciences, Farmingdale, NY, USA), DNMT1 (D63A6, #5032), PKR (N216, #2766) (Cell Signaling Technology Japan, Tokyo, Japan), phospho-PKR (E120, ab32036, abcam, Tokyo, Japan), or glyceraldehyde 3-phosphate dehydrogenase (GAPDH) (#MAB374, Millipore, Temecula, CA, USA) antibodies as an internal control, and then with peroxidase-conjugated secondary antibodies (GE Healthcare Japan, Tokyo, Japan). The bound antibodies were detected using an ECL western blotting detection system (GE Healthcare Japan).

### siRNA and transfection

The siRNA against DNMT1 (HSC.RNAI.N001130823.12.2) and negative control (DS NC1) were purchased from Integrated DNA Technologies (Coralville, IA, USA). Transient siRNA transfection in HepG2 cells was performed according to the Lipofectamine RNAiMAX (Invitrogen, Life Technologies Japan, Tokyo, Japan) reverse transfection method. In brief, 6 pmol siRNA and 1 μL Lipofectamine RNAiMAX were mixed in 100 μL Opti-MEM (Invitrogen) and added into a well in a 24-well culture plate. 5 × 10^4^ cells were seeded into a well containing siRNA and Lipofectamin reagent and cultured for 72 h.

### Construction of DNMT1-targeted shRNA expressing plasmids

Human DNMT1-specific shRNA were designed as 81-mers each containing a hairpin-loop (bold font) flanked by siRNAs (underlined) and cloned into the pBAsi-hU6-Neo vector (TAKARA), which uses the U6 RNA polymerase promoter and selective neomycin-resistant gene. The double-stranded oligodeoxyribonucleotides were synthesized as follows: 5′-GATCCGTGAGTGGAAATTAAGACTTTATGTA**CTGTGAAGCCACAGATGGG**TACATAAAGTCTTAATTTCCACTCACTTTTTTA-3′ and 5′-AGCTTAAAAAAGTGAGTGGAAATTAAGACTTTATGTA**CCCATCTGTGGCTTCACAG**TACATAAAGTCTTAATTTCCACTCACG-3′. The oligos consisted of 25 nucleotides (underlined) derived from the DNMT1 gene (Genebank accession number NM_001130823). The DNMT1 target sequences were subjected to NCBI Blast query to confirm the lack of homology to other known genes. The oligonucleotides were annealed and the annealed DNAs were ligated into the BamH I and Hind III sites of the pBAsi-hU6-Neo plasmid to create the new recombinant plasmid pBAsi-hU6-Neo-shDNMT1.

### Exposure of cells to acetaminophen or aflatoxin B1

HepG2 cells were exposed to 0.5 mM or 1.0 mM zebularine. After three days of culture, cells were re-immersed in a culture medium containing the indicated concentrations of acetaminophen (APAP) or aflatoxin B1 with zebularine, then cultured for an additional 72 h. 1 M APAP and 25 mM aflatoxin B1 were each dissolved in DMSO as a stock solution. The final DMSO concentration was equal for each drug concentration: 1.2% DMSO in APAP and 0.4% DMSO in aflatoxin B1. Cell viability after exposure to APAP or aflatoxin B1 was determined using a Cell Counting Kit-8 (Dojindo Laboratories, Kumamoto, Japan). In cultures with or without zebularine, cell cultures exposed to 0 mM APAP or 0 μM aflatoxin B1 were considered 100% viable. The cell viability of each drug-treated sample was presented as a percentage of the viability of cultures treated with 0 mM APAP or 0 μM aflatoxin B1.

### Stable transfection

The pBAsi-hU6-Neo-shDNMT1 was transfected into HepG2 cells by mixing with Lipofectamine LTX reagent (Invitrogen) according to the manufacturer’s instructions. Twenty-four hours after transfection, cells were passaged; starting the next day, they were cultured in DMEM medium containing 10% fetal bovine serum and 1000 μg/ml G418 (Roche Diagnostics K. K., Tokyo, Japan). G418-resistant clones were identified, selected and cultured, and, after three weeks of cloning, we obtained three cell lines (shDNMT1-HepG2-2, 5 and 6 cells) with stable expression of pBAsi-hU6-Neo-shDNMT1.

### Statistics

Values are expressed as means ± SEM except as noted. Statistical analyses were performed using an unpaired Student’s t-test or one-way factorial analysis of variance (ANOVA) followed by post-hoc analysis, and Tukey’s multiple comparison test. *P* < 0.05 was considered to indicate statistical significance.

### Ethics statement

All methods using human samples were performed in accordance with Ethical Guidelines for Medical and Health Research Involving Human Subjects established by the Ministry of Education, Culture, Sports, Science and Technology, and the Ministry of Health, Labour and Welfare, Japan. The entire experimental protocol using human samples was approved by the Ethical and Research Committee of the National Center for Child Health and Development. Cryopreserved primary human hepatocytes were purchased from vendors permitted to sell products derived from human organs procured in the United States of America by federally designated Organ Procurement Organizations.

## Additional Information

**How to cite this article:** Nakamura, K. *et al*. Zebularine upregulates expression of CYP genes through inhibition of DNMT1 and PKR in HepG2 cells. *Sci. Rep.* **7**, 41093; doi: 10.1038/srep41093 (2017).

**Publisher's note:** Springer Nature remains neutral with regard to jurisdictional claims in published maps and institutional affiliations.

## Supplementary Material

Supplementary Information
