# Supplementary material for: Zebularine upregulates expression of CYP genes through inhibition of DNMT1 and PKR in HepG2 cells
Source: Sci Rep. 2017 Jan 23;7:41093. doi: 10.1038/srep41093 (PMC5253741; doi:10.1038/srep41093)
Supplement: Supplementary Information [file srep41093-s1.pdf]

Supplementary Information

Zebularine upregulates expression of CYP genes through inhibition of DNMT1 and PKR in HepG2 cells

Kazuaki Nakamura[1,*], Kazuko Aizawa[1], Kyaw Htet Aung[1], Junji Yamauchi[1], and Akito Tanoue[1]

[1]Department of Pharmacology, National Research Institute for Child Health and Development, 2-10-1 Okura, Setagaya, Tokyo, 157-8535, Japan

Supplementary Table S1. TaqMan Probe Information

Gene name	Gene Description	TaqMan Assay Number
**HPRT1**	hypoxanthine phosphoribosyltransferase 1	Hs02800695_m1
**CYP1A1**	Cytochrome P450, family 1, subfamily A, polypeptide 1	Hs00153120_m1
**CYP1A2**	Cytochrome P450, family 1, subfamily A, polypeptide 2	Hs01070371_g1
**CYP2A6**	Cytochrome P450, family 2, subfamily A, polypeptide 6	Hs00868409_s1
**CYP2B6**	Cytochrome P450, family 2, subfamily B, polypeptide 6	Hs04183483_g1
**CYP2C9**	Cytochrome P450, family 2, subfamily C, polypeptide 9	Hs02383631_s1
**CYP2C19**	Cytochrome P450, family 2, subfamily C, polypeptide 19	Hs00426380_m1
**CYP2D6**	Cytochrome P450, family 2, subfamily D, polypeptide 6	Hs02576168_g1
**CYP2E1**	Cytochrome P450, family 2, subfamily E, polypeptide 1	Hs00559366_g1
**CYP3A4**	Cytochrome P450, family 3, subfamily A, polypeptide 4	Hs00604506_m1

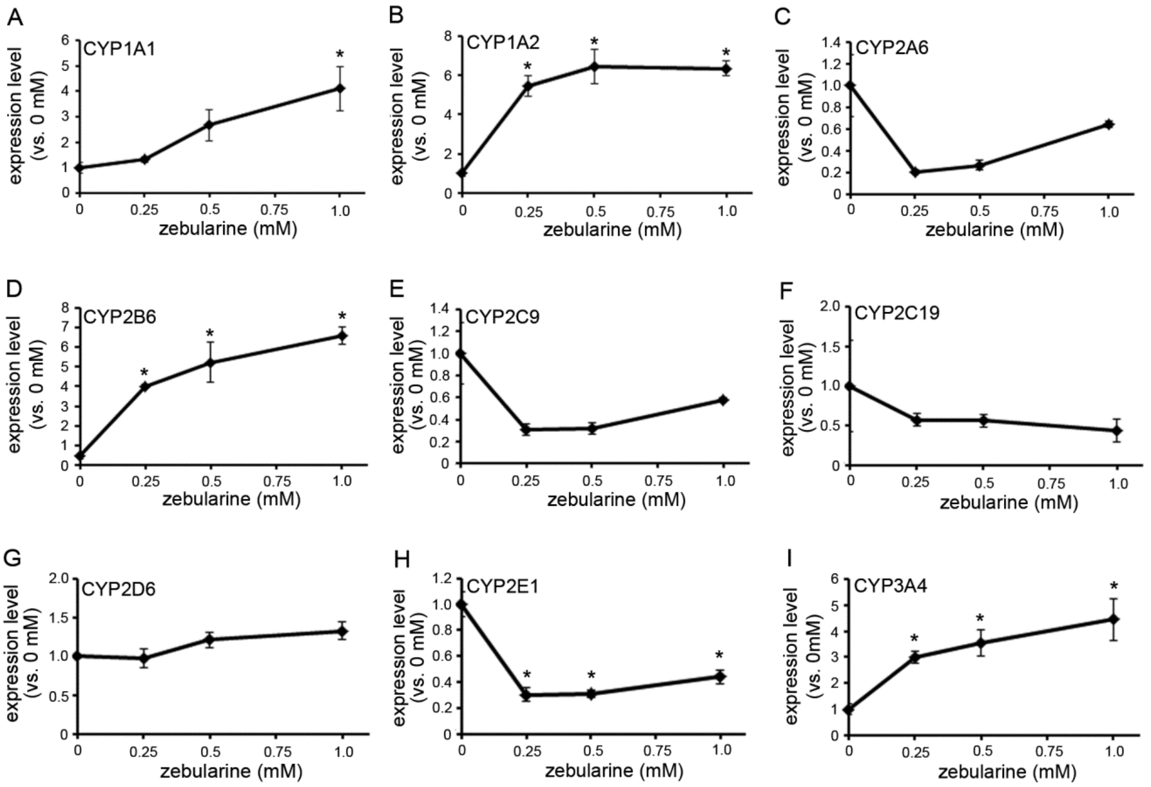

Supplementary Figure S1. The effective duration of zebularine on CYP gene expression in HepG2 cells. (**a-i)** HepG2 cells were exposed to zebularine for 72 h, after which zebularine was removed from the culture media for an additional 72 h. Expression levels of CYP1A1 (**a**), 1A2 (**b**), 2A6 (**c**), 2B6 (**d**), 2C9 (**e**), 2C19 (**f**), 2D6 (**g**), 2E1 (**h**) and 3A4 (**i**) were examined by qRT-PCR. The comparative threshold cycle (Ct) method was used to determine the relative ratio of expression for each gene, which was corrected against HPRT1. Each data point represents the mean ± SEM of the values obtained from three independent experiments. The expression level measured after exposure to 0 mM zebularine was considered to be 1.0 expression. *$p < 0.05$, compared to 0 mM.

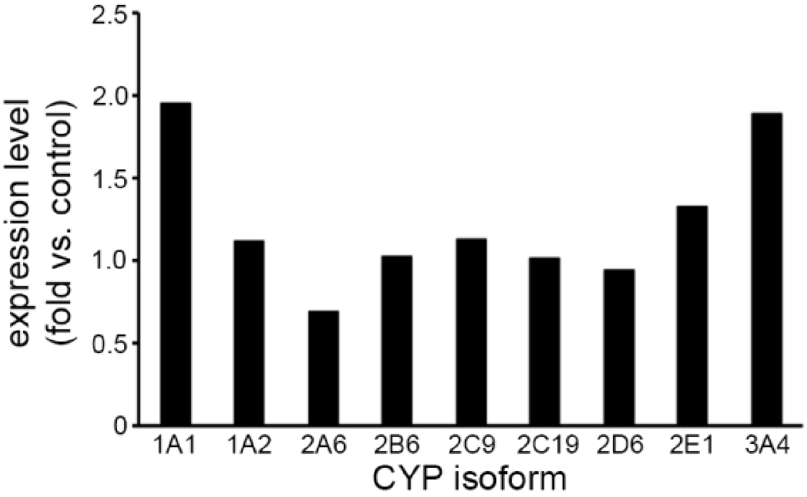

Supplementary Figure S2. The effect of zebularine on CYP gene expression in primary human hepatocytes. Primary human hepatocytes were exposed to 0.5 mM zebularine for 72 h after 96 h seeding. Expression levels of CYP1A1, 1A2, 2A6, 2B6, 2C9, 2C19, 2D6, 2E1 and 3A4 were examined by qRT-PCR. The comparative threshold cycle (Ct) method was used to determine the relative ratio of expression for each gene, which was corrected against HPRT1. Each data point represents the mean of the values obtained from triplicate analysis using one donor's hepatocytes. For each gene, the expression level detected in cells treated with 0 mM zebularine was considered to be 1.0 expression.

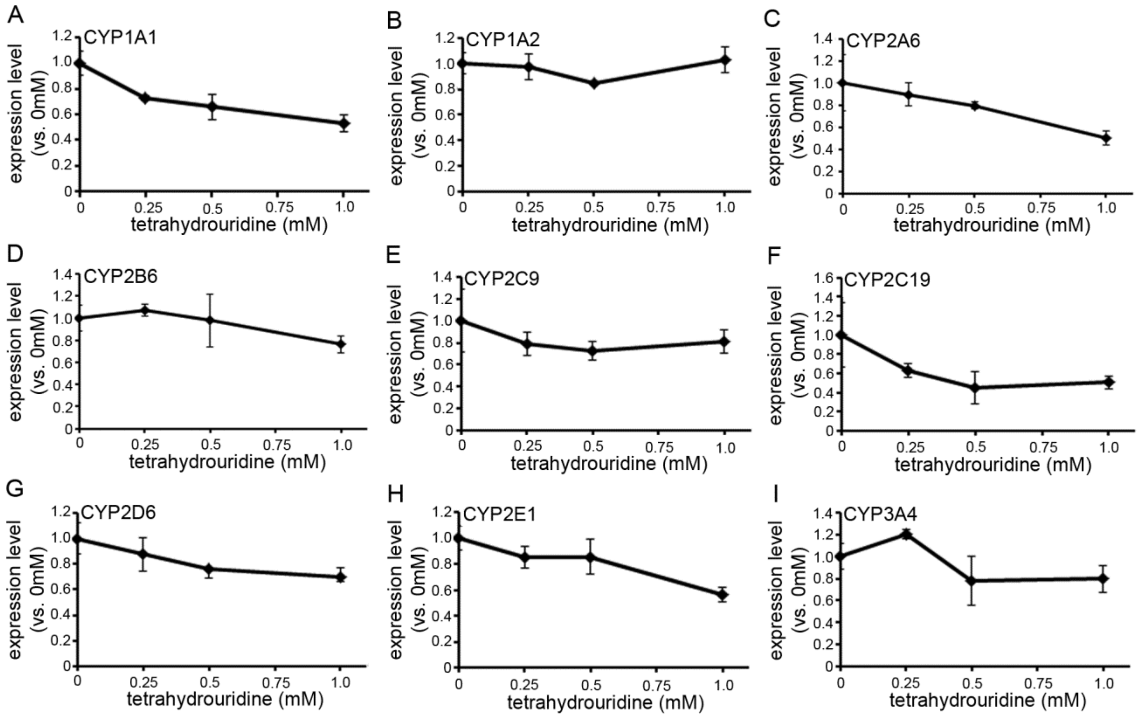

Supplementary Figure S3. The effect of tetrahydrouridine on CYP gene expression in HepG2 cells. (**a-i)** HepG2 cells were exposed to tetrahydrouridine for 72 h. Expression levels of CYP1A1 (**a**), 1A2 (**b**), 2A6 (**c**), 2B6 (**d**), 2C9 (**e**), 2C19 (**f**), 2D6 (**g**), 2E1 (**h**) and 3A4 (**i**) were examined by qRT-PCR. The comparative threshold cycle (Ct) method was used to determine the relative ratio of expression for each gene, which was corrected against HPRT1. Each data point represents the mean ± SEM of the values obtained from three independent experiments. For each gene, the expression level detected in cells treated with 0 mM tetrahydrouridine was considered to be 1.0 expression. *p < 0.05, compared to 0 mM.

Supplementary Materials and Methods

Primary culture of human hepatocytes

Cryopreserved human hepatocytes (Lot VUA, sex: female, age: 68 years, race: Caucasian), purchased from BioreclamationIVT (Baltimore, MD, USA), were suspended in Hepatocyte Culture Medium (CC-3198, Lonza, Walkersville, MD, USA). The hepatocytes were centrifuged (50 × g, 4°C) for 3 min and resuspended in the same medium. The cell suspensions were diluted to a final concentration of 9.6×10^5 viable cells/ml using Hepatocyte Culture Medium containing 2% FBS, and inocula of 4.8×10^5 viable cells/0.5 ml/well were introduced into 24 well plates that had been coated with type I collagen. The cells were cultured for 4 h after inoculation under 5% CO2 and 95% air at 37°C. The medium was then replaced with fresh medium without serum, and the cells were cultured for four days. Cells were immersed in a culture medium containing the indicated concentrations of zebularine for 72 h.
